# Flash glucose monitoring system was applied to cortisol treatment for a patient with congenital adrenal hyperplasia and 17α-hydroxylase deficiency

**DOI:** 10.1186/s12902-020-00625-1

**Published:** 2020-09-21

**Authors:** Chenyu Xiang, Minmin Han, Yi Zhang, Jianhong Yin, Li’e Pei, Jing Yang, Yunfeng Liu

**Affiliations:** 1 Department of Endocrinology, First Hospital of Shanxi Medical University, Shanxi Medical University, Taiyuan, 030000 China; 2 First Clinical Medical College, Shanxi Medical University, Taiyuan, 030000 China; 3 Department of Pharmacology, Shanxi Medical University, Taiyuan, 030000 China

**Keywords:** 17α-hydroxylase deficiency, Primary aldosteronism, Misdiagnosis, Cortisol treatment, Flash glucose monitor system

## Abstract

**Background:**

Congenital adrenal hyperplasia (CAH) with 17α-hydroxylase deficiency is a rare disease; patients often require lifetime cortisol treatment. In this case report, we presented a patient with CAH and 17α-hydroxylase deficiency, who was previously misdiagnosed as having primary aldosteronism. Furthermore, the flash glucose monitoring system (FGMS) was used to ascertain a suitable cortisol therapeutic regimen for this patient.

**Case presentation:**

A 29-year-old woman presented with sex dysgenesis, hypertension and hypokalaemia. She had been diagnosed with primary aldosteronism at a local hospital. The re-measured aldosterone level in our hospital was below the normal range after antihypertensive medication adjustment, suggesting that the primary aldosteronism was a misdiagnosis. The patient was finally diagnosed as having CAH with 17α-hydroxylase deficiency according to the endocrine profile, adrenocorticotropic hormone stimulation test, and genetic analysis. Then, the patient was recommended cortisol treatment, during which the endocrine profile, blood pressure, plasma potassium level, and blood glucose level were observed to ascertain a suitable dosage. The FGMS was used to monitor blood glucose level, which indicated that the patient’s glucose metabolism was maintained normally under the final treatment dosage.

**Conclusion:**

The misdiagnosis might have been because of the effects of the antihypertension medications on aldosterone and renin levels. The final dosage of cortisol treatment achieved a normal endocrine profile, while maintaining the homeostasis of blood glucose level, plasma potassium level and blood pressure. FGMS may be an effective method to ascertain a suitable cortisol therapeutic regimen for patients with CAH and 17α-hydroxylase deficiency.

## Background

Congenital adrenal hyperplasia (CAH) is an autosomal recessive hereditary disease, which results from mutations of the genes that encode the enzymes participating in the steroidogenesis. These enzymes involving in steroidogenic process mainly include 21-hydroxylase, 11β-hydroxylase, and 17α-hydroxylase. While 21-hydroxylase deficiency is the most common cause of the CAH [1], 17α-hydroxylase deficiency accounts for very few cases [2].

17α-hydroxylase is responsible for the biosynthesis of cortisol and sex hormones; its deficiency results in deficiencies of cortisol and sex hormones, accumulation of steroid precursors proximal to the blocks, and compensatory hypersynthesis of mineralocorticoids. As a result of the mineralocorticoid effect of 11-deoxycorticosterone and corticosterone, the activity of the renin-angiotensin-aldosterone system activity is suppressed; therefore, low aldosterone level is observed in the patients with 17α-hydroxylase deficiency. Concurrently, cortisol and sex hormones deficiencies lead to increased adrenocorticotropic hormone (ACTH) and gonadotropic hormone levels via the negative feedback system. Adrenal hyperplasia occurs as a result of excessive secretion of ACTH [3].

In female patients, 17α-hydroxylase deficiency is characterized by primary amenorrhea and sexual infantilism. In those male patients, they present with female genitalia and tend to be brought up as female. Patients of both sexes present with hypertension and hypokalaemia [4]. Lifetime cortisol replacement is the recommended therapeutic regimen for these patients.

Herein, we report the case of a patient misdiagnosed with primary aldosteronism (PA), and subsequently diagnosed as having CAH with 17α-hydroxylase deficiency. Flash glucose monitoring system (FGMS, FreeStyle Libre, Abbott Laboratories) was used to observe glucose metabolism in this patient before and after cortisol treatment to ascertain a suitable dosage.

## Case presentation

A 29-year-old woman was admitted to our hospital with hypertension and hypokalaemia. In October 2018, the patient had visited her local hospital with symptoms of hypertension and fatigue. During admission, her blood pressure reached 220/120 mmHg. Immediately, antihypertension medications were prescribed, specifically, arotinolol, valsartan and amlodipine, spironolactone, and hydrochlorothiazide. Laboratory tests suggested an increased aldosterone to renin ratio (ARR) and aldosterone level (Table 1). A computed tomography scan revealed hyperplasia in the left adrenal gland. The patient was diagnosed with PA based on the hypertension, hypokalaemia, increased ARR and adrenal hyperplasia, and discharged with antihytention drugs and potassium supplementation.

**Table 1:** Comparison of two ARR evaluations

ARR evaluation	Our hospital	Local hospital	Normal range
PRA upright (ng/ml/h)	0.01	0.14	0.93–6.56
Aldosterone upright (pg/ml)	59.63	299.16	65–295
PRA recumbent (ng/ml/h)	0.01	0.10	0.05–0.79
Aldosterone recumbent (pg/ml)	40.42	230.70	59.5–173

In December 2018, the patient returned to our hospital for extensive examination. The results of the examination revealed that the patient had no secondary female sexual characteristics; the patient reported no menstruation during adolescence. Further examination revealed the patient had an infantile vulva and absence of breast development, pubic hair, or auxiliary hair. Her blood pressure was 154/98 mmHg; a medical history interrogation indicated she had suffered hypertension and hypokalaemia since she was 13 years old.

The results of subsequent laboratory panels are presented in Table 2. The corticosterone level increased remarkably, and the progesterone level was higher than the normal value; oestradiol and testosterone levels were low; and the plasma potassium concentration was approximately within the normal range for a patient treated with a potassium supplement. Considering that arotinolol, valsartan, spironolactone, and hydrochlorothiazide might affect the ARR result, we re-examined the ARR more than 14 days after the gradual withdrawal of these medications. At this point, the aldosterone level was below the normal, and the revalued ARR was in contrast to the value observed during her last hospitalization (Table 1). In addition, we assessed the patient’s adrenal function. After performing an overnight 1 mg dexamethasone suppressing test (DST), ACTH and cortisol levels were suppressed. Furthermore, an ACTH stimulation test was performed. The patient was injected intravenously with 1 μg ACTH; blood samples were collected at baseline, after 30 min, 1 h, 2 h and 3 h to measure the cortisol level. The stimulated cortisol level did not increase.

**Table 2:** Laboratory examinations in our hospital

Laboratory examinations	Measured value	Normal range
ACTH (pmol/L)	54.77	1.6–13.9
Cortisol at 8 AM (nmol/L)	216.60	171–536
Cortisol at 4 PM (nmol/L)	158.60	64–327
Cortisol at 0 AM (nmol/L)	108.40	–
24 h UFC(nmol/24 h)	137	100–379
17-OHP (ng/ml)	0.22	< 0.93
Corticosterone (μg/L)	92.20	1.3–8.20
Urinary 17-KS (mg/24 h)	< 2.0	6.0–25.0
Urinary 17OHC (mg/24 h)	11.6	2.0–10.0
Estradiol (pmol/L)	18	18.4–201
Progesterone (nmol/L)	19.65	0.159–0.401
Testosterone (nmol/L)	0.1	0.1–1.67
K^+^ (mmol/L)	3.73	3.5–4.5
Na^+^ (mmol/L)	142	137–147
Cl^−^ (mmol/L)	106.9	99–110

Based on the high levels of ACTH and corticosterone, low levels of cortisol and aldosterone, abnormal sex hormone profile, combined with the results of the ACTH stimulating test and 1 mg DST, we conceived a diagnosis of CAH with 17α-hydroxylase deficiency. Blood samples were collected for genetic analysis and informed consent was obtained from the patient and her parents. The results revealed that patient held a homozygous frame-shift mutation in her CYP17A1 gene: c.985_987delTACinsAA (p.Y329KfsX418), inherited from her parents. Karyotype analysis was identified as 46, XX.

Finally, we confirmed the diagnosis of CAH with 17α-hydroxylase deficiency based on the patient’s medical history, laboratory results, imaging findings, and the genetic analysis. We then initiated scheduled cortisol treatment. We recommended oral prednisone acetate 2.5 mg twice daily and potassium chloride 1 g four times daily to continue potassium replenishment. The patient’s normal blood pressure was maintained with the amlodipine besylate at 5 mg once daily.

During the follow-ups, laboratory tests were performed to assess therapeutic efficacy (Table 3). In the first follow-up, the recommended dosage of prednisone acetate was adjusted from 2.5 mg twice daily to 2.5 mg thrice daily. The prednisone dosage in the evening was halved in the second follow-up. The patient’s blood pressure was eventually stabilized and maintained at a desired level with amlodipine besylate 5 mg once daily, although unsatisfactory blood pressure occurred transiently during the treatment. We gradually tapered the dosage of potassium chloride and the plasma potassium level was 4.32 mmol/L (3.5–4.5 mmol/L) a week after potassium supplement suspension.

**Table 3:** Results of laboratory examinations in the follow ups

Laboratory examinations	2019.02.21	2019.03.21	2019.04.21	Normal range
PRA upright (ng/ml/h)	2.24	–	–	0.93–6.56
Aldosterone upright (pg/ml)	179.74	–	–	65–296
ACTH (pmol/L)	35.88	0.44	8.64	1.6–13.9
Progesterone (nmol/L)	17	0.16	–	0.159–0.401
Corticosterone (μg/L)	125	2.22	–	1.3–8.20
K^+^ (mmol/L)	4.06	5.07	4.69	3.5–5.5
Prednisone dosage(mg)	2.5–2.5	2.5–2.5-2.5	2.5–2.5-1.25	–

**Fig. 1:**
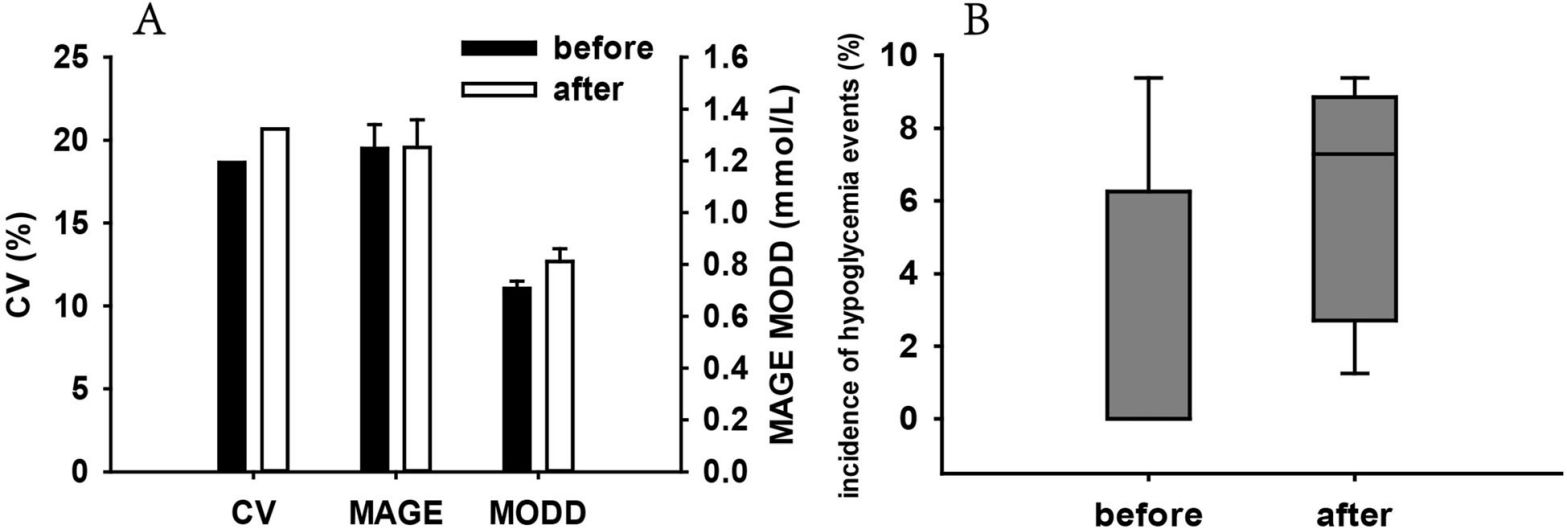
Comparison of FGMS parameters and hypoglycaemia event incidence before and after treatment. **a** CV, MAGE, MODD; **b** hypoglycaemia event incidence. CV is presented as mean; MAGE and MODD are presented as mean ± SEM; and hypoglycaemia event incidence is denoted as median with 10th, 25th,75th, and 90th percentiles

**Fig. 2:**
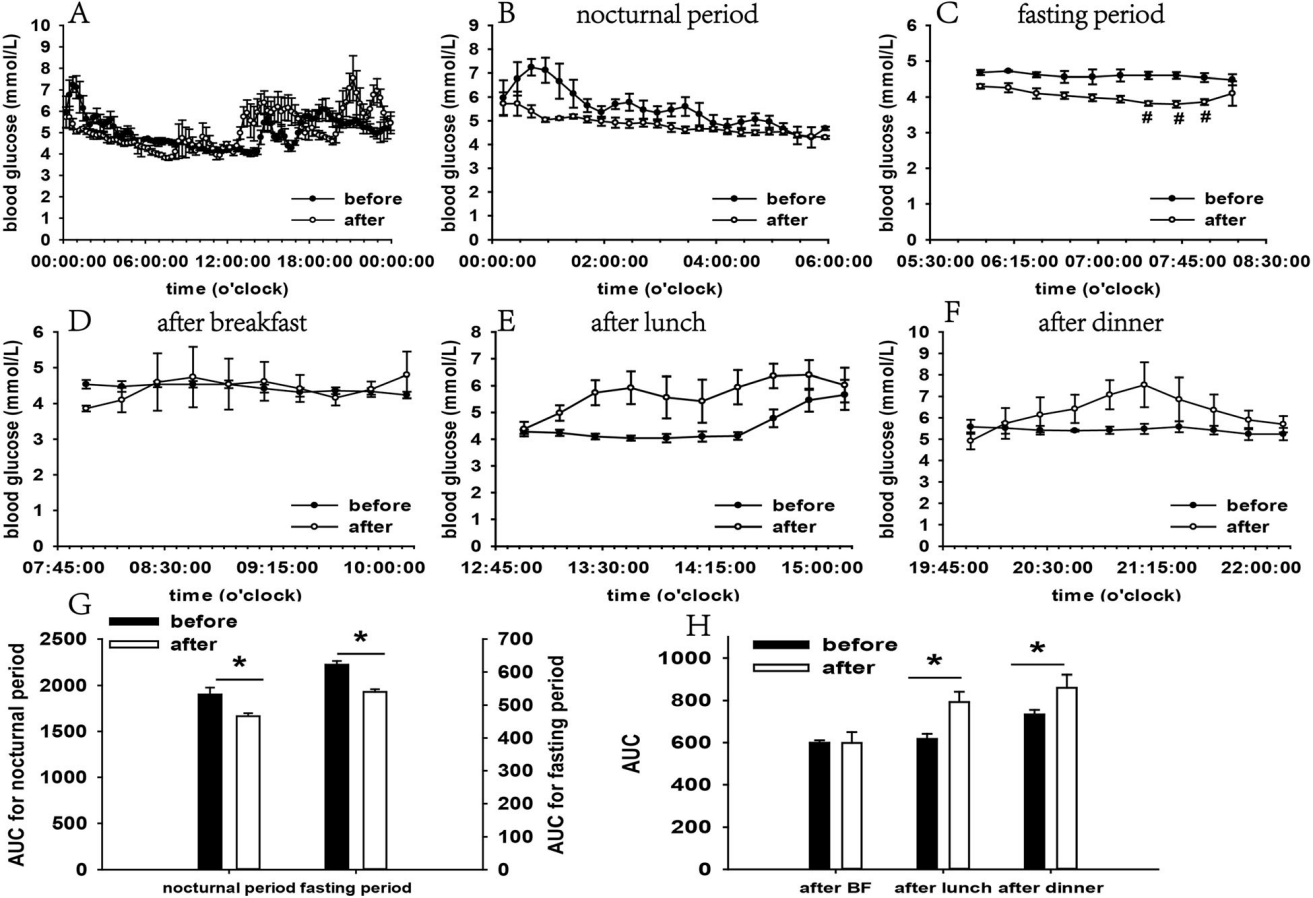
Comparison of mean blood glucose levels and AUC before and after treatment. Mealtime was presented as follows: breakfast at 8 AM; lunch at 1 PM; and dinner at 8 PM. Mean blood glucose levels: (**a**) during the day; (**b**) nocturnal period; (**c**) fasting period; (**d**) after breakfast (BF); (**e**) after lunch; and (**f**) after dinner. Mean AUC: (**g**) nocturnal and fasting periods; and (**h**) postprandial periods. The values are presented as mean ± SEM. *represented statistically different AUC at this time period (*P* < 0.05). ^#^represents hypoglycaemia (3.8–3.9 mmol/L)

More importantly, we performed sequential observation of the blood glucose level before treatment and after the third follow-up using FGMS. Five-day blood glucose data collected before and after treatment were analyzed. FGMS parameters, such as coefficient variance (CV), mean amplitude of glucose excursion (MAGE), and mean of daily differences (MODD) representing general, with-day, and day-to-day glucose variability (GV) were calculated and compared before and after treatment; no significant difference was identified in these parameters (Fig. 1). Blood glucose levels at nocturnal (0–6 AM), fasting (6–8 AM), and postprandial periods (8–10 AM, 1–3 PM, and 8–10 PM) were specifically analyzed. The overall blood glucose level was nearly within the normal range before and after treatment (Fig. 2). Nocturnal and fasting blood glucose levels decreased significantly after cortisol treatment and three hypoglycaemia points (3.82 mmol/L, 3.8 mmol/L, and 3.86 mmol/L) were identified during the fasting period (Fig. 2); the incidence of hypoglycaemia event during the monitoring before and after treatment were further compared and no significance was identified (Fig. 1). Postprandial blood glucose levels increased after treatment at 1–3 PM and 8–10 PM (Fig. 2).

## Discussion and conclusion

In this case, the patient’s previous test results at the local hospital, including increased aldosterone level and ARR suggested the diagnosis of PA. However, the ARR evaluation at the local hospital had been conducted while the medications were being administered, which likely influenced the aldosterone and renin levels [5–8]. Furthermore, the observed sexual dysgenesis and the glucocorticoid insufficiency were incompatible with the diagnosis of PA. We gradually stopped the administration of antihypertensive drugs and subsequently recommended treatment with amlodipine besylate instead [9]. Another ARR examination was conducted after a 14-day period of medication adjustment [10]; at this point, the ARR indicated decreased aldosterone level, ruling out the diagnosis of PA. The patient was finally diagnosed with CAH and 17-hydroxylase deficiency according to the endocrine profile, ACTH stimulation test, and genetic analysis.

Once CAH with 17α-hydroxylase deficiency was confirmed, we immediately recommended cortisol treatment (2.5 mg prednisone twice daily). In general, it is believed that patients with this disease should undergo lifetime cortisol treatment; however, there is no uniform recommendation on the optimal cortisol treatment dosage. In the present case, the FGMS was introduced to ascertain a suitable cortisol replacement regimen because of the effect of exogenous cortisol administration on glucose metabolism.

A previous research has investigated cortisol dose adjustment using continuous glucose monitoring in patients with secondary hypoadrenalism [11]. CAH with 17α-hydroxylase deficiency leads to adrenal insufficiency; however, incomplete cortisol deficiency was the distinguishing feature of our patient. Not only should an optimum cortisol treatment dosage suppress the mineralocorticoids synthesis to maintain normal blood pressure and plasma potassium level, but it should also supply cortisol deficiency and minimize the risk of over administration, including exogenous hypercortisolism and adrenocortical suppression. The usage of FGMS can effectively indicate a suitable treatment dosage from glucose metabolism perspective.

In the first follow-up, to adequately suppress ACTH secretion, we recommended oral administration of prednisone acetate 2.5 mg thrice daily. As a result, ACTH level decreased and its stimulating effect on adrenocortical hormone secretion was weakened. Moreover, accumulation of corticosterone, one of the mediated metabolites of the adrenocortical hormones production, was mitigated, leading, to some extent, to improvement in hypertension and hypokalaemia. Following the principle of normalizing electrolytes and hormone levels with the smallest effective dose of cortisol, we recommended a lower dose in the evening, based on the over-suppressed ACTH. The final dosage of prednisone was 2.5 mg in the morning, 2.5 mg at the midday and 1.25 mg in the evening. The patient’s blood pressure and plasma potassium were maintained at satisfactory levels with the final prednisone dosage and an extra 5 mg amlodipine besylate.

Considering FGMS monitoring data, no significant changes were identified in the GV parameters, which suggested that the glycometabolism was normal and stable during the cortisol treatment, providing evidence for the suitable prednisone dosage from the glucose fluctuation perspective. Blood glucose levels at nocturnal and fasting periods significantly decreased with three hypoglycaemia points during the fasting period after cortisol treatment. This observation was contrary to the commonly held belief that cortisol can lead to blood glucose elevation. We assumed that corticosterone’s glucocorticoid-like function accounted for higher nocturnal and fasting blood glucose levels before treatment [12]. In addition, this significant blood glucose level reduction might reflect suppressed adrenocortical function by prednisone overdose; however, no significant changes were identified after further comparison of hypoglycaemia event incidence during the FGMS monitoring before and after treatment. Normal but significantly increased postprandial blood glucose level was observed after cortisol treatment compared with that before treatment. Based on the above-mentioned results, it can be determined that cortisol treatment improved the patient’s glycometabolism condition; however, this provided insufficient evidence for a prednisone overdose. Furthermore, the patient’s endocrine profile, blood pressure, and blood potassium were maintained in the normal range and stable with the final prednisone dose. Finally, we recommended no prednisone dose adjustment and regular follow-up. Further, we will continue to pay attention to the patient’s condition and adjust the prednisone dose if necessary.

In summary, the present case study reported about a patient diagnosed with CAH and 17α-hydroxylase deficiency, previously misdiagnosed with PA because of the effect of antihypertension medications on aldosterone and renin levels. Furthermore, we recommended a suitable prednisone dosage, which achieved normal endocrine profile and homeostasis of plasma potassium, blood pressure, and blood glucose. FGMS was demonstrated as an effective method to ascertain a suitable cortisol treatment for patients with CAH and 17α-hydroxylase deficiency.

## Data Availability

All data generated and analyzed during the current study were included in this published article and available from the corresponding authors on reasonable request.

## Abbreviations

CAH: Congenital adrenal hyperplasia
PA: Primary aldosteronism
ACTH: Adrenocorticotropic hormone
ARR: Aldosterone to renin ratio
DST: Dexamethasone suppressing test
FGMS: Flash glucose monitor system
AUC: Area under curve
GV: Glucose variability
CV: Coefficient variance
MAGE: Mean amplitude of glycemic excursion
MODD: Mean of daily differences
BF: Breakfast
PRA: Plasma renin activity
24 h UFC: 24-h urinary free cortisol
17-OHP: 17α-hydroxyprogesterone
17-KS: 17-ketosterone
17OHC: 17-hydroxycorticosterone
